# Psychological factors and premenstrual syndrome: A Spanish case-control study

**DOI:** 10.1371/journal.pone.0212557

**Published:** 2019-03-06

**Authors:** María del Mar Fernández, Carlos Regueira-Méndez, Bahi Takkouche

**Affiliations:** 1 Department of Preventive Medicine, University of Santiago de Compostela, Santiago de Compostela, Spain; 2 Centro de Investigación Biomédica en Red de Epidemiología y Salud Pública (CIBER-ESP), Madrid, Spain; Ordu University, TURKEY

## Abstract

**Objective:**

To assess whether the psychological variables perceived stress, neuroticism and coping strategies, are associated with Premenstrual Syndrome (PMS) and Premenstrual Dysphoric Syndrome (PMDD).

**Design:**

Case-control study with incident cases using the Spanish public healthcare system.

**Setting:**

3 major public hospitals and one family counseling and planning center.

**Population:**

Women consulting for troubles related to menstruation and for other motives such as screening for uterine cancer, contraception counselling or desire for pregnancy.

**Methods:**

Logistic regression.

**Main outcome measures:**

Odds of PMS and PMDD.

**Results:**

285 PMS and 285 age-matched controls, as well as 88 PMDD cases and 176 controls participated in the study. Medium and high levels of perceived stress were associated with an increase in the odds of PMS (Odds Ratio (OR) = 2.49; 95%CI: 1.41–4.39 and OR = 4.90; 95%CI: 2.70–8.89, respectively). For PMDD the results were: OR = 2.61; 95%CI: 1.35–5.05 and OR = 5.79; 95%CI: 2.63–12.76, respectively.

Subjects with medium and high levels of neuroticism were also at higher odds of suffering from PMS (OR = 2.53; 95%CI: 1.06–6.06 and OR = 8.05; 95%CI: 3.07–2.12, respectively). For PMDD, the results were OR = 3.70; 95%CI: 1.27–10.77 and 5.73: 95%CI: 1.96–16.77, respectively.

High levels in the large majority of coping strategies were also associated with increased odds of PMS and PMDD.

**Conclusions:**

Psychological factors including perceived stress, neuroticism and coping strategies are strongly related to PMS/PMDD. This association is unlikely to be due to confounding or misclassification bias. A reverse causation process cannot be ruled out although its likelihood is remote.

## Introduction

Premenstrual syndrome (PMS) and its most severe form, Premenstrual Dysphoric Disorder (PMDD), are defined as the set of recurrent physical and psychic symptoms in the luteal phase of the menstrual cycle[1]. Although classified separately, both clinical groups have a common pathophysiological basis[2].

While the prevalence of PMS varies between 20% and 40%, that of PMDD sways between 3 and 8% in women of childbearing age in the United States.[3] Worldwide, according to a recent meta-analysis, about half of women suffer from this syndrome.[4]

PMS affects the daily life of women as it interferes with work, study, and interpersonal relationships[3,5]. The annual total costs per woman in the US were estimated at $ 5,000[5]. A large proportion of cases is not diagnosed due to the difficulties encountered by the physicians in establishing a diagnosis and to the fact that women do not usually consult a doctor when they present symptoms of PMS[6]. These difficulties often relate to gender bias. Indeed, part of the community of health professionals is reluctant to diagnose a syndrome that they consider a mere cultural and social construct and not a real disease[7].

As a result, despite the high frequency of the syndrome, the burden of women seeking medical help and receiving a diagnosis is small and is probably declining[8]. It is remarkable that, before receiving a diagnosis, these women, on average, had sought medical help for 5.33 years from 3.75 physicians[9].

Previous studies have related PMS/PMDD and psychological and personality factors, such as psychological stress, coping styles and neuroticism[10–16]. However, these studies often use a cross-sectional design which hinders any causal inference.

Although disagreement exists about the meaning of the term “stress”, a common core concept refers to a process in which external demands (also called stressors) exceed the adaptation capacity of the organism and cause undesirable psychological and biological changes[17]. To cope with stress, several styles are used, including positive reframing, religious behavior or even substance use. These coping strategies are linked to personality types such as neuroticism. Neuroticism is a stable personality trait that can be defined as the individual’s inadequate response to stress and is characterized by instability and emotional insecurity, high levels of anxiety, along with psychosomatic symptoms[18]. It has been shown that subjects with a high degree of neuroticism tend to use avoidance or support coping strategies[19].

The objective of this study is, therefore, to investigate whether perceived stress, coping styles and neurotic personality are associated with premenstrual syndrome.

## Methods

### Study population

We set up a case-control study with newly diagnosed cases of PMS and PMDD. We selected 285 consecutive cases of PMS among women consulting for troubles related to menstruation and 285 controls. Motives of consultation included: heavy menstrual bleeding, hypomenorrhea, irregular menstruation cycles, amenorrhea and dysmenorrhea. We also selected 88 cases of PMDD and 176 controls. Participants were selected from three major public hospitals and one family counseling and planning center in the city of Santiago de Compostela and surroundings (Northwest Spain), which attend a population of approximately 400,000 users. Controls were individually age-matched to cases in each center.

All participants signed a consent form. The study was approved by Comité Ético de Investigación Clínica de Galicia n°2011/117, PI: Carlos Regueira-Méndez.

### Data collection

We collected the data through an anonymous, voluntary and self-completed questionnaire. Participants were selected by gynecologists and midwives of each center.

The questionnaire included a series of items about potential confounders of the relationship between psychological and personality variables and PMS, such as symptoms related to menstruation, socio-demographic factors, constitutional factors and other lifestyle factors.

To determine cases, we used the PSST (Premenstrual Syndrome Screening Tool) questionnaire[20], which consists of 19 questions about physical, behavioral and psychological symptoms in the five days before menstruation of the previous three months. The severity of each symptom was graded from 1 to 4 (1: none, 4: severe). The score obtained from this questionnaire, together with the presence of specific symptoms such as irritability and nervousness were used to differentiate between PMS and PMDD.

To define a PMS or PMDD case, the following algorithm was used. First, to label a case as PMS, we required a score ≥ 3 in one of the four questions about whether the women felt "irritable", "tense", "tearful" or "depressed". To define a PMDD case, we required a score of 4 in one of these same four questions. Furthermore, to define a case of PMS, a score ≥ 3 in one of the five variables of interference with "work performance, relationship with colleagues, family members, in social life or in household tasks" was required. Again, this score had to be 4 to define a case of PMDD. The last condition to be met to define PMS and PMDD cases is a score ≥ 3 in at least four of the first 14 questions (i.e. all questions except the five questions related to interference). Patients that did not meet the requirements were excluded from the study.

Controls were selected from women who attended the same health facility as cases but for motives different from PMS such as screening for uterine cancer, contraception counselling or desire for pregnancy. We confirmed the absence of PMS using the same questionnaire as above. The controls were required to have a score ≤2 in each item that described whether they felt "irritable", "tense", "tearful" or "depressed" and also a score ≤ 2 on all the interference variables described above.

We measured psychologic stress during the last three months by means of the scale of perceived stress proposed by Cohen et al. and validated in a Spanish population [21].

To assess coping styles, we used the scale proposed by Carver et al.[22] which differentiates 14 types of coping, each of them evaluated by two items graded from 0 to 3. Additionally, we computed an average coping score by adding the scores of the 28 items divided by 28.

The degree of neuroticism was measured using 12 items of the scale proposed by Costa and McCrae (60 items)[23].

### Statistical analysis

We used conditional logistic regression to obtain crude and adjusted Odds Ratios and their corresponding 95% confidence intervals. We conducted separate analyses for PMS and PMDD. Continuous variables that did not follow a normal distribution were log-transformed and those variables the distribution of which still showed important asymmetry after logarithmic transformation were divided into quartiles or terciles, according to the range of their distribution.

In the final model, those variables that modified the estimate of the OR of psychological variables by more than 10% were introduced[24]. These variables had shown previously some association with the risk of PMS in the univariate analysis. The final model included the following variables: number of hours of sleep, satisfaction with the quality of sleep and age of menarche.

The following candidate variables were analyzed for potential inclusion in the model but were finally discarded: menstrual irregularity, dysmenorrhea, number of pregnancies, antecedents of abortions, use of anovulants and intrauterine device, treatment with antidepressants, caffeine intake, alcohol intake, intake of various nutrients, and total energy expenditure.

The analyses were performed with STATA version 12[25].

## Results

We included 285 cases with PMS and 285 age-matched controls (case-control ratio 1:1), as well as 88 cases with PMDD and their 176 corresponding age-matched controls (case-control ratio 1:2). The response rate was 80% among initially approached cases and 80% in controls. The average age was 32 years for cases and controls, both for PMS and PMDD. Due to the low number of partial missing data, and thus, the fact that these missing data could not sensibly modify the results, we did not perform any imputation procedure.

In Table 1 we observe that PMS cases had an earlier menarche and a lower body mass index than the control group, as well as a higher proportion of nulliparous women. Due to the fact that the city in which this case-control study was carried out is a university city, a large proportion of the sample had high educational level. PMS cases had higher education than controls. In Table 2, this imbalance is also observed between PMDD cases and their controls.

**Table 1:** Distribution of 285 PMS cases and 285 age-matched controls according to social, anthropometric and gynecological variables.

Characteristics	Category	N° cases	%	N° controls	%
**Body mass index (Kg/m**^**2**^)*	<19	22	8.1	*14*	*5*.*2*
	19–24.9	182	67.4	*160*	*59*.*9*
	25–30	41	15.2	*62*	*23*.*2*
	>30	25	9.3	*31*	*11*.*6*
Educational level*	primary	46	16.3	*46*	*16*.*1*
	secondary	80	28.4	*111*	*38*.*9*
	university	156	55.3	*128*	*44*.*9*
Menarche age*	< = 11	74	26.1	*48*	*16*.*8*
	12 y 13	153	54.1	*162*	*56*.*8*
	> = 14	56	19.8	*75*	*26*.*3*
**Number of pregnancies**	0	147	51.6	*131*	*46*.*0*
	1	66	23.2	*70*	*24*.*6*
	> 1	72	25.3	*84*	*29*.*5*
**Number of abortions**	0	233	81.8	*229*	*80*.*4*
	1	43	15.1	*49*	*17*.*2*
	> 1	9	3.2	*7*	*2*.*5*
Oral contraception*	no	181	80.8	*187*	*81*.*0*
	yes	43	19.2	*44*	*19*.*0*
Intrauterine device*	no	235	92.9	*240*	*91*.*3*
	yes	18	7.1	*23*	*8*.*7*

*The sum of different categories is < 285 due to partial missing data

**Table 2:** Distribution of 88 PMDD cases and 176 age-matched controls according to social, anthropometric and gynecological variables.

Characteristics	Category	N° cases	%	N° controls	%
Body mass index (Kg/m2)*	<19	8	9.6	*9*	*5*.*4*
	19–24.9	56	67.5	*108*	*65*.*1*
	25–30	14	16.9	*35*	*21*.*1*
	>30	5	6.0	*14*	*8*.*4*
Educational level*	primary	18	20.9	*31*	*17*.*6*
	secondary	23	26.7	*72*	*40*.*9*
	university	45	52.3	*73*	*41*.*5*
**Menarche age**	<= 11	21	23.9	*27*	*15*.*3*
	12 y 13	50	56.8	*98*	*55*.*7*
	>= 14	17	19.3	*51*	*29*.*0*
**Number of pregnancies**	0	47	53.4	*74*	*42*.*0*
	1	18	20.5	*48*	*27*.*3*
	> 1	23	26.1	*54*	*30*.*7*
**Number of abortions**	0	69	78.4	*147*	*83*.*5*
	1	13	14.8	*24*	*13*.*6*
	> 1	6	6.8	*5*	*2*.*8*
Oral contraception*	no	57	80.3	*112*	*80*.*0*
	yes	14	19.7	*28*	*20*.*0*
Intrauterine device*	no	72	93.5	*147*	*93*.*0*
	yes	5	6.5	*11*	*7*.*0*

*The sum of different categories is < the total number of cases or controls due to partial missing data

### Effect of psychosocial and personality variables on PMS

The data of the relation between PMS and psychosocial and personality variables are shown in Table 3.

**Table 3:** Crude and adjusted Odds Ratios of perceived stress and neuroticism.

Variable	Category	N° cases	%	N° controls	%	Crude OR	Adjusted OR*
**PMS**							
Perceived stress	1st quartile	53	19.1	103	37.6	1	1
	2nd quartile	73	26.4	84	30.7	1.58 (0.98–2.56)	1.62 (0.91–2.69)
	3rd quartile	55	19.9	48	17.5	2.25 (1.32–3.84)	2.49 (1.41–4.39)
	4th quartile	96	34.7	39	14.5	4.69 (2.71–8.11)	4.90 (2.70–8.89)
Neuroticism	1st quartile	43	17.3	75	31.9	1	1
	2nd quartile	57	22.9	85	36.2	1.06 (0.59–1.90)	0.97 (0.53–1.80)
	3rd quartile	82	32.9	52	22.1	2.83 (1.52–5.26)	2.61 (1.35–5.05)
	4th quartile	67	26.9	23	9.8	4.74 (2.34–9.60)	5.79 (2.63–12.76)
**PMDD**							
Perceived stress	1st quartile	16	18.8	65	38.2	1	1
	2nd quartile	8	9.4	40	23.5	0.78 (0.29–2.12)	0.83 (0.29–2.32)
	3rd quartile	23	27.1	42	24.7	2.59 (1.12–6.00)	2.53 (1.06–6.06)
	4th quartile	38	44.7	23	13.5	8.93 (6.63–21.98)	8.05 (3.07–2.12)
Neuroticism	1st quartile	10	12.7	42	27.6	1	1
	2nd quartile	18	22.8	56	36.8	1.46 (0.54–3.97)	1.80 (0.64–5.06)
	3rd quartile	21	26.6	24	15.8	3.58 (1.29–9.98)	3.70 (1.27–10.77)
	4th quartile	30	38.0	30	19.7	5.75 (2.08–15.84)	5.73 (1.96–16.77)

* Adjusted for sleep hours, sleep satisfaction and age at menarche

Medium and high levels (3^rd^ and 4^th^ quartiles of the distribution) of perceived stress are strongly associated with PMS: OR = 2.49, 95% CI: 1.41–4.39 and OR = 4.90, 95% CI: 2.70–8.89, respectively. Furthermore, medium and high levels of neuroticism exert a similar effect on PMS (OR = 2.61, 95% CI: 1.35–5.05 and OR = 5.79; 95% CI: 2.63–12.76 respectively).

Table 4 shows that high levels of total coping score (4^th^ quartile) are also related to PMS (OR = 3.26; 95% CI: 1.70–6.24). Furthermore, high levels of the following coping strategies were associated with a considerable increase in the odds of PMS: “planning” (OR = 2.17; 95% CI: 1.19–3.96), “use of emotional support” (OR = 2.06; 95% CI: 1.16–3.66), “use of instrumental support” (OR = 2.14; 95% CI: 1.24–3.70), “self-distraction” (OR = 2.39; 95% CI: 1.35–4.23), “venting” (OR = 2.56; 95% CI: 1.37–4.79), behavioural disengagement (OR = 2.58; 95% CI: 1.16–5.75), and self-blame (OR = 4.57; 95% CI: 2.40–8.73). Substance use was also strongly associated with PMS (OR = 2.50; 95% CI: 1.46–4.28).

**Table 4:** Crude and adjusted Odds Ratios of coping styles and PMS.

Variable	Category	N° cases	%	N° controls	%	Crude OR	Adjusted OR*
Total coping	1st quartile	58	21.7	79	30.2	1	1
	2nd quartile	72	27.0	83	31.7	1.20 (0.72–2.02)	1.06 (0.61–1.86)
	3rd quartile	57	21.3	62	23.7	1.25 (0.73–2.13)	1.26 (0.71–2.22)
	4th quartile	80	30.0	38	14.5	2.92 (1.61–5.29)	3.26 (1.70–6.24)
Active coping	1st quartile	57	20.7	51	26.7	1	1
	2nd quartile	71	25.8	78	21.8	1.56 (0.94–2.59)	1.63 (0.95–2.81)
	3rd quartile	84	30.5	56	28.6	1.47 (0.89–2.43)	1.70 (0.99–2.91)
	4th quartile	63	22.9	61	22.9	1.18 (0.69–2.01)	1.37 (0.77–2.42)
Planning	1st quartile	80	29.0	101	37.8	1	1
	2nd quartile	59	21.4	59	22.1	1.32 (0.81–2.13)	1.32 (0.80–2.20)
	3rd quartile	76	27.5	68	25.5	1.50 (0.92–2.44)	1.52 (0.90–2.57)
	4th quartile	61	22.1	39	14.6	2.11 (1.21–3.68)	2.17 (1.19–3.96)
Positive reframing	1st quartile	21	7.6	17	6.4	1	1
	2nd quartile	87	31.4	91	34.1	0.81 (0.40–1.63)	0.88 (0.42–1.85)
	3rd quartile	58	20.9	56	21.0	0.83 (0.40–1.73)	0.99 (0.45–2.18)
	4th quartile	111	40.1	103	38.6	0.89 (0.44–1.81)	1.00 (0.48–2.12)
Acceptance	1st quartile	82	29.9	73	27.3	1	1
	2nd quartile	66	24.1	59	22.1	1.00 (0.61–1.63)	0.95 (0.56–1.60)
	3rd quartile	70	25.5	71	26.6	0.86 (0.54–1.40)	0.82 (0.49–1.38)
	4th quartile	56	20.4	64	24.0	0.74 (0.44–1.22)	0.82 (0.48–1.42)
Humor	1st quartile	74	26.8	66	24.7	1	1
	2nd quartile	101	36.6	107	40.1	0.85 (0.54–1.33)	0.90 (0.55–1.45)
	3rd quartile	23	8.3	31	11.6	0.60 (0.31–1.14)	0.60 (0.30–1.20)
	4th quartile	78	28.3	63	23.6	1.05 (0.66–1.67)	1.20 (0.73–1.97)
Religion	1st quartile	134	48.6	139	51.9	1	1
	2nd quartile	47	47.0	35	13.1	1.56 (0.93–2.63)	1.66 (0.95–2.89)
	3rd quartile	58	58.0	66	24.6	0.90 (0.59–1.37)	1.00 (0.63–1.57)
	4th quartile	37	37.0	28	10.4	1.47 (0.83–2.59)	1.37 (0.74–2.55)
Using emotional support	1st quartile	115	41.7	129	48.3	1	1
	2nd quartile	55	19.9	56	21.0	1.11 (0.70–1.75)	1.11 (0.68–1.81)
	3rd quartile	54	19.6	54	20.2	1.05 (0.66–1.66)	1.25 (0.73–2.04)
	4th quartile	52	18.8	28	10.5	2.07 (1.19–3.59)	2.06 (1.16–3.66)
Using instrumental support	1st quartile	114	41.2	131	49.2	1	1
	2nd quartile	44	15.9	50	18.8	1.03 (0.63–1.69)	1.09 (0.64–1.84)
	3rd quartile	59	21.3	50	18.8	1.42 (0.89–2.28)	1.53 (0.93–2.53)
	4th quartile	60	21.7	35	13.2	1.99 (1.18–3.57)	2.14 (1.24–3.70)
Self-distraction	1st quartile	102	37.0	127	47.6	1	1
	2nd quartile	67	24.3	77	28.8	1.08 (0.69–1.69)	1.17 (0.72–1.89)
	3rd quartile	56	20.3	38	14.2	1.61 (0.97–2.68)	1.69 (0.99–2.91)
	4th quartile	51	18.5	25	9.4	2.27 (1.32–3.91)	2.39 (1.35–4.23)
Denial	1st quartile	102	36.8	124	46.4	1	1
	2nd quartile	70	25.3	63	23.6	1.37 (0.89–2.10)	1.44 (0.91–2.27)
	3rd quartile	73	26.4	58	21.7	1.61 (1.02–2.54)	1.67 (1.02–2.72)
	4th quartile	32	11.6	22	8.2	2.02 (1.06–3.88)	1.7 (0.85–3.39)
Venting	1st quartile	46	16.7	57	21.5	1	1
	2nd quartile	85	30.8	100	37.7	0.97 (0.60–1.57)	1.14 (0.68–1.91)
	3rd quartile	75	27.2	65	24.5	1.42 (0.83–2.41)	1.38 (0.79–2.42)
	4th quartile	70	25.4	43	16.2	2.19 (1.23–3.92)	2.56 (1.37–4.79)
Substance use	no	219	79.1	241	90.3	1	1
	yes	58	20.9	26	9.7	2.43 (1.46–4.04)	2.50 (1.46–4.28)
Behavioral disengagement	1st quartile	112	40.6	137	51.7	1	1
	2nd quartile	75	27.2	67	25.3	1.42 (0.89–2.55)	1.47 (0.91–2.40)
	3rd quartile	56	20.3	46	17.4	1.47 (0.91–2.38)	1.52 (0.90–2.56)
	4th quartile	33	12.0	15	5.7	3.1 (1.47–6.56)	2.58 (1.16–5.75)
Self-Blame	1st quartile	58	21	91	34.1	1	1
	2nd quartile	83	30.1	92	34.5	1.42 (0.88–2.30)	1.61 (0.96–2.71)
	3rd quartile	47	17	47	17.6	1.55 (0.89–2.72)	1.63 (0.96–2.94)
	4th quartile	88	31.9	37	13.9	4.11 (2.29–7.38)	4.57 (2.40–8.73)

*Adjusted for sleep hours, sleep satisfaction and age at menarche

### Effect of psychosocial and personality variables in PMDD

Globally, the results for PMDD resemble those obtained for PMS (Table 3). Medium and high levels (3^rd^ and 4^th^ quartiles of the distribution) of perceived stress are associated with PMDD: OR = 2.53, 95% CI: 1.06–6.06 and OR = 8.05, 95% CI: 3.07–21.12, respectively. Furthermore, medium and high levels of neuroticism exert a similar effect on PMDD (OR = 3.70, 95% CI: 1.27–10.77 and OR = 5.73; 95% CI: 1.96–16.77 respectively).

High levels of total coping score (4^th^ quartile) are related to the odds of PMDD (OR = 3.94; 95% CI: 1.58–9.79) (Table 5). Among the coping styles that showed an effect, medium levels of “active coping”, but not high levels, are associated with PMS (OR = 3.18; 95% CI: 1.23–8.20). Other coping strategies the high levels of which were strongly associated with PMDD were as follows: “planning” (OR = 3.98; 95% CI: 1.53–10.35), “use of instrumental support” (OR = 2.61; 95% CI: 1.11–6.15), “self-distraction” (OR = 4.58; 95% CI: 1.80–11.64), “venting” (OR = 3.17; 95% CI: 1.34–7.46), behavioural disengagement (OR = 5.06; 95% CI: 1.46–17.53), and self-blame (OR = 5.88; 95% CI: 2.43–14.20). As observed for PMS, substance use was also strongly associated with PMDD (OR = 5.95; 95% CI: 2.39–14.83).

**Table 5:** Crude and adjusted Odds Ratios of coping styles and PMDD.

Variable	Category	N° cases	%	N° controls	%	Crude OR	Adjusted OR*
Total coping	1st quartile	18	22.0	56	34.8	1	1
	2nd quartile	13	15.9	39	24.2	1.06 (0.46–2.41)	1.26 (0.51–3.13)
	3rd quartile	21	25.6	42	26.1	1.45 (0.66–3.16)	1.52 (0.61–3.75)
	4th quartile	30	36.6	24	14.9	4.03 (1.76–9.23)	3.94 (1.58–9.79)
Active coping	1st quartile	57	20.7	71	26.7	1	1
	2nd quartile	71	25.8	58	21.8	2.92 (1.21–7.07)	4.33 (1.56–12.00)
	3rd quartile	84	30.5	76	28.6	2.12 (0.93–4.81)	3.18 (1.23–8.20)
	4th quartile	63	22.9	61	22.9	1.51 (0.64–3.56)	1.83 (0.72–4.70)
Planning	1st quartile	18	22.0	68	41.2	1	1
	2nd quartile	23	28.0	32	19.4	2.62 (1.20–5.72)	2.65 (1.13–6.20)
	3rd quartile	22	26.8	45	27.3	2.04 (0.95–4.41)	2.14 (0.90–5.09)
	4th quartile	19	23.2	20	12.1	3.46 (1.45–8.26)	3.98 (1.53–10.35)
Positive reframing	1st quartile	21	7.6	17	6.4	1	1
	2nd quartile	87	31.4	91	34.1	1.04 (0.34–3.19)	1.27 (0.37–4.31)
	3rd quartile	58	20.9	56	21.0	0.84 (0.27–2.62)	0.85 (0.25–2.89)
	4th quartile	111	40.1	103	38.6	1.12 (0.38–3.34)	1.39 (0.42–4.60)
Acceptance	1st quartile	82	29.9	73	27.3	1	1
	2nd quartile	66	24.1	59	22.1	1.44 (0.68–3.07)	1.55 (0.67–3.58)
	3rd quartile	70	25.5	71	26.6	1.51 (0.71–3.22)	2.12 (0.91–4.94)
	4th quartile	56	20.4	64	24.0	0.87 (0.39–1.91)	0.98 (0.40–2.39)
Humor	1st quartile	74	26.8	66	24.7	1	1
	2nd quartile	101	36.6	107	40.1	0.87 (0.43–1.76)	0.98 (0.46–2.10)
	3rd quartile	23	8.3	31	11.6	1.29 (0.49–3.36)	1.37 (0.49–3.85)
	4th quartile	78	28.3	63	23.6	1.27 (0.61–2.64)	1.34 (0.59–3.00)
Religion	1st quartile	134	48.6	139	51.9	1	1
	2nd quartile	47	17.0	35	13.1	1.89 (0.87–4.01)	1.75 (0.77–3.98)
	3rd quartile	58	21.0	66	24.6	0.78 (0.39–1.57)	0.83 (0.39–1.76)
	4th quartile	37	13.4	28	10.4	1.42 (0.60–3.36)	1.05 (0.40–2.77)
Using emotional support	1st quartile	115	41.7	129	48.3	1	1
	2nd quartile	55	19.9	56	21.0	1.23 (0.60–2.50)	1.25 (0.57–2.72)
	3rd quartile	54	19.6	54	20.2	1.11 (0.55–2.27)	1.63 (0.72–3.67)
	4th quartile	52	18.8	28	10.5	2.10 (0.89–4.95)	2.04 (0.81–5.11)
Using instrumental support	1st quartile	35	42.2	85	51.8	1	1
	2nd quartile	11	13.3	32	19.5	0.81 (0.37–1.80)	0.75 (0.30–1.85)
	3rd quartile	19	22.9	29	17.7	1.64 (0.80–3.34)	2.33 (1.03–5.26)
	4th quartile	18	21.7	18	11.0	2.42 (1.09–5.37)	2.61 (1.11–6.15)
Self-distraction	1st quartile	23	27.7	71	43.0	1	1
	2nd quartile	18	21.7	50	30.3	1.22 (0.58–2.57)	1.11 (0.49–2.48)
	3rd quartile	18	21.7	24	14.5	1.98 (0.90–4.39)	2.18 (0.91–5.18)
	4th quartile	24	28.9	20	12.1	4.73 (2.00–11.15)	4.58 (1.80–11.64)
Denial	1st quartile	102	36.8	124	46.4	1	1
	2nd quartile	70	25.3	63	23.6	1 (0.49–2.07)	0.88 (0.41–1.91)
	3rd quartile	73	26.4	58	21.7	1.35 (0.70–2.63)	1.52 (0.72–3.19)
	4th quartile	32	11.6	22	8.2	2.64 (0.97–7.18)	1.60 (0.52–4.96)
Venting	1st quartile	18	21.7	48	29.3	1	1
	2nd quartile	19	22.9	56	34.1	0.66 (0.29–1.49)	0.65 (0.27–1.61)
	3rd quartile	18	21.7	38	23.2	0.97 (0.43–2.22)	0.79 (0.31–2.01)
	4th quartile	28	33.7	22	13.4	2.73 (1.31–5.69)	3.17 (1.34–7.46)
Substance use	no	59	71.1	153	92.7	1	1
	yes	24	28.9	12	7.3	5.94 (2.54–13.92)	5.95 (2.39–14.83)
Behavioral disengagement	1st quartile	27	32.9	88	53.7	1	1
	2nd quartile	23	28.0	41	25.0	1.74 (0.86–3.52)	1.51 (0.72–3.16)
	3rd quartile	19	23.2	26	15.9	2.61 (1.15–5.96)	2.28 (0.95–5.46)
	4th quartile	13	15.9	9	5.5	6.58 (2.14–20.26)	5.06 (1.46–17.53)
Self-Blame	1st quartile	20	24.1	61	37.0	1	1
	2nd quartile	18	21.7	56	33.9	1.03 (0.49–2.14)	1.11 (0.50–2.94)
	3rd quartile	14	16.9	30	18.2	1.47 (0.63–3.44)	1.54 (0.61–3.86)
	4th quartile	31	37.3	18	10.9	5.02 (2.29–11.04)	5.88 (2.43–14.2)

*Adjusted for sleep hours, sleep satisfaction and age at menarche

## Discussion

Our findings indicate that high levels of psychological stress and neuroticism are strongly associated with PMS and PMDD. While the effect of high levels of neuroticism is similar in PMS and PMDD, that of perceived stress is considerably stronger in PMDD.

High levels of coping styles are also related to PMS and PMDD. Except for 4 out of 14 styles (positive reframing, acceptance, humor and turning to religion), PMS and PMDD cases were more susceptible to present high coping scores than controls. Carver et al. classify coping strategies in two broad patterns: “adaptive coping” which includes active coping, planning, positive reframing, acceptance, emotional and instrumental support; and “dysfunctional coping” which includes denial, behavioral disengagement, venting, substance use and self-distraction[22]. The rest of coping styles was not explicitly assigned to one or another group. Our analysis did not find evidence of differences in the effect between the two coping patterns in PMS or PMDD.

Our results are in accordance with the several studies carried out on psychological factors and the development of PMS[10–16]. The large majority of these studies used a cross-sectional design and thus, are not optimal for causal inference purposes.

The effect observed in our study is not easily ascribed to confounding as a large set of potential confounders were considered in the analysis and estimates were not significantly modified when these potential confounders were introduced in the model.

The general view that is generally accepted is that psychological factors interact with physiological menstrual cycle changes and symptoms to produce distress. Some women respond to these cycle changes using dysfunctional coping. This maladaptation increases the premenstrual symptoms that are eventually labeled as PMS[26]. Indeed, coping with PMS may be rendered extremely difficult due to male-centered values, behaviors and attitudes toward women suffering from this syndrome[27].

PMS is due to a hypersensitivity of the woman to changes in the activity of gonadal hormones and its development possibly involves complex interactions of hormonal, neural, and behavioral factors[28,29]. It has been suggested that the main etiological factor of this hypersensitivity is a genetically-determined predisposition. More specifically, holders of a polymorphism in the serotonin transporter promoter gene (5-HTTLPR) have been associated with a higher risk of PMS[16,30]. This genetic factor, not assessed in our study, could possibly play the role of effect modifier in the relation between psychological factors and PMS/PMDD. The effect of stress and neuroticism could then be different depending on whether women harbor this polymorphism or do not.

In our study, we cannot rule out some amount of misclassification in the PMS/PMDD assessment since the diagnosis is based on a subjective symptom score. Furthermore, this assessment, as in any case-control study, was carried out retrospectively. Some authors have suggested that retrospective assessment of PMS could potentially cause overreport of symptom severity, which could lead to the inclusion of subjects without true PMS in the case group[8]. However, this misclassification bias, if any, is unlikely to modify the conclusion of this study because if there is any erroneous classification, it is likely that this error occurs regardless of the exposure status, since the subjects were not aware of the hypothesis of the study that related psychological factors and PMS. To explore the direction and magnitude of the potential bias due to misclassification of outcome, we reanalyzed the data by introducing the stress and neuroticism exposure variables as dichotomous (exposed/non-exposed). The resulting Odds Ratios were for PMS/perceived stress: 2.68 (95% CI: 1.78–4.06), PMS/neuroticism 3.61 (95% CI: 2.19–5.94), PMDD/perceived stress 4.34 (95% CI: 2.12–8.88), and PMDD/neuroticism 3.14 (95% CI: 1.62–6.08). These results suggest that the true association between these psychological factor and PMS may even be stronger than the one we have observed, as non-differential misclassification of a dichotomous variable always yield bias towards the null value, i.e decreases the effect.

Our study was a case-control study in which cases of PMS/PMDD were incident. Theoretically, the levels of perceived stress, neuroticism and coping refer to a time window that precedes the onset of the syndrome. However, it is not unlikely that the premenstrual symptoms, albeit in a less intense form, were concomitant to the assessment of psychological factors. A reverse causation process, in which the presence of premenstrual symptoms produces stress and inadequate coping strategies, cannot be ruled out. This could explain the fact that high levels of certain adaptive coping strategies, expected to reduce the odds of PMS/PMDD, were eventually associated with a large increase in the odds risk.

Furthermore, as in any case-control study, our study may be subject to recall bias. PMS/PMDD cases may better assess their psychological factors than controls. However, this is unlikely to occur as the participants were not aware of the hypothesis of the study. Indeed, the hypothesis of a relation between psychological factors and premenstrual syndrome was not disclosed to the participants, as these factors were only part of a long list of exposure factors that have been assessed in the questionnaire.

## Conclusion

This case-control study found a strong association between psychological factors including perceived stress, neuroticism and coping strategies and the occurrence of PMS/PMDD. The association persisted after control for several risk factors and is unlikely to be due to misclassification bias. Although from a strict point of view a reverse causation process cannot be ruled out, due to the nature of the exposure factors explored, our findings are strengthened by the fact that they are in general agreement with previous work and by the biological plausibility of the relation between psychological stress and PMS/PMDD.

Future research on the etiology of PMS should abandon cross-sectional designs as suggested by PMS experts a decade ago[31]. Indeed, this type of design does not allow for adequate causal inference, a fact that may render any finding deceptive. Stress reduction programs may be an effective prevention tool as recommended by experts[32]. It has been shown that, in the long run, non-avoidant coping, also called attention coping, was associated with more positive outcomes. However, avoidant tactics may be more effective in the short run[33]. Last, serious efforts should be made by the community of health professionals to modify prevailing cultural attitudes and overcome gender-biased detrimental decisions in the diagnosis and control of premenstrual syndrome.

## Supporting information

S1 File Questionnaire: Original Spanish Version. (PDF)

S2 File Questionnaire: English translation. (DOCX)

S3 File Database PMS. (DTA)

S4 File Database PMDD. (DTA)

S5 File Variables definition. (ODT)
