# Supplementary material for: Psychological factors and premenstrual syndrome: A Spanish case-control study
Source: PLoS One. 2019 Mar 6;14(3):e0212557. doi: 10.1371/journal.pone.0212557 (PMC6402625; doi:10.1371/journal.pone.0212557)
Supplement: S1 File — (PDF) [file pone.0212557.s001.pdf]

ESTUDIO DE LOS FACTORES DE RIESGO DE LA DISMENORREA Y DEL SÍNDROME PREMENSTRUAL

INSTRUCCIONES
1. En las preguntas abiertas ponga un dígito en cada casilla.
2. En las preguntas multiopción llene el círculo sin salirse de él.

Correcto: ● ⊗ *Incorrecto:* ⌀ ⊠

PACIENTE

fecha nacimiento | | | | | | | edad menarquia | | | peso (Kg) | | | talla (cm) | | |

profesión ______________________

	no tiene	primarios	medios	superiores
estudios	○	○	○	○

	0	1	2	>2
embarazos	○	○	○	○
abortos	○	○	○	○

(para las no embarazadas)

	ahora	antes	nunca	... desde	< 3 meses	3 - 12 meses	1 a 2 años	> 2 años
toma anovulatorios	○	○	○	... desde	○	○	○	○
¿es portadora de DIU?	○	○	○	... desde	○	○	○	○

SÍNTOMAS

	mucha	bastante	poca	nada
¿qué regularidad tienen sus menstruaciones?	○	○	○	○

	mucho menor	menor	igual	mayor	mucho mayor
¿el dolor de sus menstruaciones varió después de tomar anovulatorios?	○	○	○	○	○

¿durante los días previos y durante la regla tiene estos síntomas?

los 5 días antes de la regla...	en la última regla 1	2	3	4	habitualmente 1	2	3	4
...se siente irritable, enfadada	○	○	○	○	○	○	○	○
...tensa, con ansiedad	○	○	○	○	○	○	○	○
...llorosa, emotiva	○	○	○	○	○	○	○	○
...deprimida, hundida	○	○	○	○	○	○	○	○
...menos interés en actividades domésticas	○	○	○	○	○	○	○	○
...Ídem en actividades laborales	○	○	○	○	○	○	○	○
...Ídem en actividades sociales	○	○	○	○	○	○	○	○
...dificultad para concentrarse	○	○	○	○	○	○	○	○
...se siente cansada o apática	○	○	○	○	○	○	○	○
...come más, tiene antojos	○	○	○	○	○	○	○	○
...duerme menos de los normal	○	○	○	○	○	○	○	○
...suele dormir más de lo normal	○	○	○	○	○	○	○	○
...se siente abrumada, sin control	○	○	○	○	○	○	○	○
¿tuvo 1 ó más de los siguientes síntomas: pechos sensibles, dolor de cabeza o muscular, vientre hinchado o sintió como si ganara peso?	○	○	○	○	○	○	○	○
¿algún problema anterior **interfirió** en su rendimiento laboral?	○	○	○	○	○	○	○	○
...en su relación con sus compañeros?	○	○	○	○	○	○	○	○
...en su relación con sus familiares?	○	○	○	○	○	○	○	○
...en su vida social?	○	○	○	○	○	○	○	○
...en las tareas de su hogar?	○	○	○	○	○	○	○	○

desde el 1er día de la regla...	en la última regla 1	2	3	4	5	habitualmente 1	2	3	4	5
...tiene retortijones abdominales	○	○	○	○	○	○	○	○	○	○
...tiene dolor intermitente	○	○	○	○	○	○	○	○	○	○
... se acurruca en cama con algo caliente en el vientre	○	○	○	○	○	○	○	○	○	○
...tiene más doloridas las zonas lumbar y abdominal	○	○	○	○	○	○	○	○	○	○
...tiene dolor de espalda	○	○	○	○	○	○	○	○	○	○
...tiene náuseas	○	○	○	○	○	○	○	○	○	○

durante la regla...	en la última regla 1	2	3	4	5	habitualmente 1	2	3	4	5
...tiene dolor sordo, no intenso	○	○	○	○	○	○	○	○	○	○
...toma aspirina para el dolor	○	○	○	○	○	○	○	○	○	○
...toma otro medicamento	○	○	○	○	○	○	○	○	○	○
...se siente débil o mareada	○	○	○	○	○	○	○	○	○	○
...tiene diarrea	○	○	○	○	○	○	○	○	○	○
...tiene estreñimiento	○	○	○	○	○	○	○	○	○	○

	en la última regla: no tuvo	<1	1	2	>2	habitualmente: no tiene	<1	1	2	>2
...el dolor dura (días)...	○	○	○	○	○	○	○	○	○	○
...el dolor le incapacita en su actividad diaria (días)	○	○	○	○	○	○	○	○	○	○

	nada	algo	bastante	mucho	nada	algo	bastante	mucho
¿el dolor **interfirió** en su rendimiento laboral?	○	○	○	○	○	○	○	○
...en su vida social?	○	○	○	○	○	○	○	○
...en las tareas del hogar?	○	○	○	○	○	○	○	○

	escasa	moderada	abundante	escasa	moderada	abundante
...la hemorragia es...	○	○	○	○	○	○

COMORBILIDADES

*¿ha sido diagnosticada de alguna de las siguientes enfermedades? Si fue así ¿**a qué edad** comenzó?* (puede cubrir más de una opción)

	diagnosis médica sin HC	diagnosis médica en HC	edad
ovario poliquístico	○	○	| | |
endometriosis	○	○	| | |

MEDICAMENTOS

*¿durante los últimos 3 meses ha tomado al menos **3 veces por semana** algún medicamento?* (incluidos complejos vitamínicos)

______________________	| | |	○ ○ ○	| | |
______________________	| | |	○ ○ ○	| | |
______________________	| | |	○ ○ ○	| | |
______________________	| | |	○ ○ ○	| | |

CONSUMO DE TABACO

	no	sí
*¿ha fumado de manera regular (al menos 1 cigarrillo, puro o pipa al día) **durante más de 6 meses**?*	○	○

	con filtro	sin filtro	edad la 1ª vez	edad última vez	nº	al día	a la semana
rubio	○	○	| | |	| | |	| | |	○	○
negro	○	○	| | |	| | |	| | |	○	○
puros/pipas	○	○	| | |	| | |	| | |	○	○

	0	1	2	3	4	>4
nº personas que fuman en casa	○	○	○	○	○	○

	<1	1ó2	3ó4	5ó6	7u8	>8
¿cuántas horas pasa a la semana en lugares en los que la gente fuma?	○	○	○	○	○	○

EJERCICIO FÍSICO

	mucho menor	menor	igual	mayor	mucho mayor
su actividad física comparando con otras personas de su misma edad es...	○	○	○	○	○

	<5	6-15	16-30	31-45	>45
¿cuántos minutos dedica al día a caminar o ir en bicicleta al trabajo o de compras?	○	○	○	○	○

en su tiempo libre...	nunca	raras veces	a veces	a menudo	muy a menudo
... pasea en bicicleta	○	○	○	○	○
... practica un deporte	○	○	○	○	○
... ve la televisión	○	○	○	○	○
... pasea caminando	○	○	○	○	○
... suele hacer algo que le haga sudar	○	○	○	○	○

si suele practicar algunos deportes o ir al gimnasio, indique la intensidad que requieren y el tiempo que les dedica:

	pequeña	media	grande
intensidad (1er deporte)	○	○	○
intensidad (2º deporte)	○	○	○

pequeña: andar; media: ciclismo, gimnasia, natación; grande: fútbol

nº horas semanales que dedica a 1er deporte | | | ...a 2º deporte | | |

nº meses al año que dedica a 1er deporte | | | ...a 2º deporte | | |

CONFIDENCIAL
NO DIFUNDIR, NI EN PARTE NI EN SU TOTALIDAD,
SIN AUTORIZACIÓN DE LOS AUTORES

SUEÑO

	<= 6	7	8	9	> 9
¿cuántas horas acostumbra a dormir por la noche?	O	O	O	O	O
	nada	poco	regular	bastante	mucho
¿en qué grado está satisfecho con la calidad de su sueño?	O	O	O	O	O
	0	1-15	16-30	31-45	>46
¿cuántos minutos suele dormir de siesta?	O	O	O	O	O

CARÁCTER

¿En qué medida está de acuerdo con las siguientes frases?

Grado de acuerdo: A: en desacuerdo total
B: bastante en desacuerdo C: neutral
D: bastante de acuerdo E: totalmente de acuerdo

NO VISUALIZABLE POR MOTIVO DE DERECHO DE AUTOR

A	B	C	D	E
O	O	O	O	O
O	O	O	O	O
O	O	O	O	O
O	O	O	O	O
O	O	O	O	O
O	O	O	O	O
O	O	O	O	O
O	O	O	O	O
O	O	O	O	O
O	O	O	O	O
O	O	O	O	O
O	O	O	O	O

ESTRÉS PERCIBIDO

*durante los **últimos 3 meses** globalmente Vd. diría que se ha sentido identificado con las frases siguientes:*

	nada	algo	regular	bastante	mucho
incapaz de controlar cosas importantes en su vida	O	O	O	O	O
sin confianza para manejar sus problemas personales	O	O	O	O	O
que las cosas no le van bien	O	O	O	O	O
con tantos problemas que se sintió sobrepasado	O	O	O	O	O

AFRONTAMIENTO

Indique qué hace y qué siente ante ellos	nunca	a veces	a menudo	muy a menudo
me oriento hacia el trabajo o a otras actividades para distraer mi mente	O	O	O	O
concentro mis esfuerzos en hacer algo sobre la situación en la que estoy	O	O	O	O
me digo a mí mismo/a que "esto no es real"	O	O	O	O
utilizo el alcohol u otras drogas para sentirme mejor	O	O	O	O
intento obtener el apoyo emocional de los demás	O	O	O	O
me doy por vencido a la hora de lidiar con ello	O	O	O	O
tomo medidas para hacer que la situación sea mejor	O	O	O	O
me niego a creer lo que ha sucedido	O	O	O	O
me digo a mí mismo/a cosas para dejar que los sentimientos desagradables se escapen	O	O	O	O
busco ayuda y consejo de los demás	O	O	O	O
consumo alcohol u otras drogas para que me ayuden a ir tirando	O	O	O	O
intento verlo desde otro ángulo para hacer que las cosas parezcan más positivas	O	O	O	O
me critico a mí mismo/a	O	O	O	O
intento elaborar una estrategia sobre qué hacer	O	O	O	O
Intento conseguir el apoyo y la comprensión de alguien	O	O	O	O
renuncio a hacerle frente a la situación	O	O	O	O
busco algo bueno en lo que ha sucedido	O	O	O	O
hago bromas sobre ello	O	O	O	O
hago algo para pensar menos en eso, como ir al cine, ver la TV, leer, dormir ...	O	O	O	O
acepto la realidad de que eso haya sucedido	O	O	O	O
expreso mis sentimientos negativos	O	O	O	O
intento buscar apoyo en la religión o en mis creencias espirituales	O	O	O	O
intento que otras personas me ayuden o me aconsejen sobre qué hacer	O	O	O	O
aprendo a vivir con ello	O	O	O	O
pienso mucho en cuáles son los pasos a seguir	O	O	O	O
me culpo a mí mismo/a por las cosas que suceden	O	O	O	O
rezo o medito	O	O	O	O
bromeo sobre la situación	O	O	O	O

OBSERVACIONES

CONFIDENCIAL
NO DIFUNDIR, NI EN PARTE NI EN SU TOTALIDAD,
SIN AUTORIZACIÓN DE LOS AUTORES
