# Supplementary material for: Psychological factors and premenstrual syndrome: A Spanish case-control study
Source: PLoS One. 2019 Mar 6;14(3):e0212557. doi: 10.1371/journal.pone.0212557 (PMC6402625; doi:10.1371/journal.pone.0212557)
Supplement: S2 File — (DOCX) [file pone.0212557.s002.docx]

**Study of risk factors of dysmenorrhea and premenstrual syndrome**

**Patient identification**

Birthdate

Age of menarche

Weight

Height

Educational level: *none, primary, college,university.*

Occupation:

Numbers of pregnancies: *0 1 2 >2*

Number of abortions: *0 1 2 >2*

**For non-pregnant women:**

Do you or did you take the contraceptive pill?

*Now before never*

For how long?

*<3 months 3-12 months 1-2 years > 2 years*

Do you or did you use an IUD ?

*now before never*

For how long?

*<3 months 3-12 months 1-2 years > 2 years*

**Symptoms**

How regular are your menses?

*very regular/ quite regular/ rather irregular/ not regular at all*

Did you regularly have your last 3 menses?

*Yes/ no*

How did your menstrual discomfort change after getting pregnant?

*much less /less/ did not change/ stronger/ much stronger*

And after taking the contraceptive pill?

*much less /less/ did not change/ stronger/ much stronger*

**Within 5 days before you have your menses do you have these symptoms?**

***[in the last menstruation / usually]***

…feels irritable / angry

… tense / anxious

… tearful / with increases sensitivity to rejection

…depressed/hopeless

.. less interested in work activities

.. less interested in home activities

.. less interested in social activities

…difficulty in concentrating

… with fatigue / lack of energy

… overeating / food cravings

… sleeps less than ususal

… sleeps more than usual

…feels overwhelmed, out of control

… with breast tenderness, headaches, joint or muscle pain, bloating, weight gain

Did the symptoms above interfere with?

…your work efficiency

…your relationships woth coworkers

…your relationship with your family

…your social life

…your home responsibilities

**From the first day of the period:**

…you have cramps in your abdomen

…you have intermittent pain

…you curl up in bed, using something hot on your abdomen,

… you feel more pain in the low back and in in the abdomen

… you have back pain,

… you feel nauseous

During your period:

… you have a dull ache, not intense

… you take aspirin to mitigate the pain

… you take any other medicine

… you feel weak or dizzy

… you have diarrhea

… you are constipated

.. The pain lasts for:  *<1 day / 1 day / 2days/ >2 days*

… The pain makes you unable to do your daily activity for: *<1 day / 1 day / 2days / >2 days*

… The pain interfered in your work efficiency: *<1 day / 1 day / 2days/ >2 days*

… The pain interfered in your social life: *<1 day / 1 day / 2days/ >2 days*

… The pain interfered in your house work: *<1 day / 1 day / 2days/ >2 days*

The bleeding is: *scarce / moderate / abundant*

**Comorbidities**

Have you been diagnosed with any of the following diseases? If so, at what age did it start? (you can select more than one option)

[Diagnosis without clinical chart / Diagnosis with clinical chart]

Polycystic ovary syndrome:

Endometriosis:

**Medicines**

During the last three months, have you taken any medications (including vitamin supplements at least 3 times a week)?

*….*

*….*

*….*

**Smoking**

Have you ever smoked regularly (at least 1 cigarette, cigar or pipe per day) **for more than 6 months**?

*No*

*Yes*

*Per day / Per week*

*With filter / without filter*

*Age at first time / age at last time*

number of people who smoke at home?

*0 / 1 / 2 / 3 / 4 / > 4*

How many hours per week do you spend in places where people smoke?

*<1 / 1-2 / 3-4 / 5-6 / 7-8 / > 8*

**Physical exercise**

Compared to other people of the same age, your physical activity is:

*much less intense / less intense / has the same intensity / more intense / much more intense*

How many minutes/day do you spend walking or cycling to go to work or shopping ?

*<5 / 6-15 / 16-30 / 31-45 / > 45*

In your free time…

*never / rarely / sometimes / quite often / very often*

... you ride a bike

… you practice some sport

… you watching television

… you go for a walk

… you do an activity that makes you sweat

If you usually practice a sport or go to the gym, please mention the intensity this activity demands from you and the time you spend doing this activity.

 Intensity (first sport): *low / moderate / high*

Intensity (second sport): *low / moderate / high*

*(low intensity: walking; moderate: cycling, fitness, swimming; high: football)*

Number of hours per week you practice: first sport / second sport

Number of months per year you practice: first sport / second sport

**Sleep**

How many hours do you usually sleep at night?

*<= 6 / 7 / 8 / 9 / > 9*

How satisfied you are with the quality of your sleep

*not satisfied at all/ a little satisfied /somewhat satisfied / rather satisfied / very satisfied*

How many minutes do you usually have a nap?

*0 / 1-15 / 16-30 / 31-45 / > 45*

**Personality**

Please mention your degree of agreement with the following sentences

*completely disagree / disagree / neutral / agree / strongly agree*

***NOT AVAILABLE. INFORMATION PROTECTED BY COPYRIGHT***

**Perceived stress**

In the the last 3 months globally you would say that you felt that:

*not at all / sometimes / moderately / quite a lot / very often.*

… you were unable to control the important things in your life

… you were not confident about your ability to handle your personal problems

… things were not going well

… that there were so many difficulties that you could not overcome them?

**Coping**

Please tell what you do to cope with stressful events.

*Never / sometimes / often / very often*

I turn to work or other activities to distract my mind

I concentrate my efforts on doing something about the situation I am in.

I tell myself that this is not real

I use alcohol or other drugs to feel better;

I try to get emotional support from others

I give up trying to deal with it

I take measures to make the situation better

I refuse to believe what has happened

I say things to let unpleasant feelings escape

I seek help and advice from others

I use alcohol or other drugs to help me get through it

I try to see it from another angle to make things look more positive

I criticize myself

I try to elaborate a strategy about what to do

I try to get comfort and understanding from someone

I give up the attempt to cope

I look for something good in what has happened

I make jokes about it

I do something to think about it less, such as go to the movies, watch TV, read, or sleep

I accept the reality of the fact that this has happened

I express my negative feelings

I try to find support in religion or in my spiritual beliefs

I try that other people help me or advise me about what to do

I learn to live with it

I think hard about what steps to take

I blame myself for the things that happened

I pray or meditate

I make fun of the situation

**Comments**

| \|  \| \| --- \| |  |  |
| --- | --- | --- | --- |
