# Supplementary material for: Psychological factors and premenstrual syndrome: A Spanish case-control study
Source: PLoS One. 2019 Mar 6;14(3):e0212557. doi: 10.1371/journal.pone.0212557 (PMC6402625; doi:10.1371/journal.pone.0212557)
Supplement: Supplementary file 3 [file pone.0212557.s005.odt]

Men:	Age at menarche:  (< 11=1)  (12 - 13=2)  (>14=3)					

edadT:	age							

pms/pmdd:	case definition (0: control, 1: case)						

pareado_PMS/PMDD:	matching variable							

strspcb: perceived stress							

coping:	total score coping							

cpt: different coping styles as per Carver							

neurot: neuroticism							

nhs3: sleep hours 1=7-8; 2=8;·3=>8							

sats3: sleep satisfaction 1= not satisfied or little satisfied; 2=moderately satified;3= satisfied or very satisfied							

estud3: educational level: (1=primary school)(2=college)(3=university)  (1=1)  (2=1)  (3=2)  (4=3)  							

nembzi: nº pregnancies: (1=0)  (2=1)  (3=2) // (0=none) (1= one) (2= 2 or more)			

naborti: nº  abortions:  (0 = none) (1 = one) (2= 2 or more)					

anoviact: current oral contraceptive use (0=no; 1=yes) 						

diuiact:	current IUD use: ( 0=NO)(1=yes)    							

BMI4c:	Body Mass Index: (< 19=1)  (19 -24.9=2)  (25 - 30=3)  (>30 =4)
